# 1582. Real-World Use of Long-Acting Cabotegravir + Rilpivirine in the US: Effectiveness in the First Year

**DOI:** 10.1093/ofid/ofac492.105

**Published:** 2022-12-15

**Authors:** Michael G Sension, Ricky K Hsu, Jennifer S Fusco, Laurence Brunet, Quateka Cochran, Christine Uranaka, Gayathri Sridhar, Vani Vannappagari, Andrew Zolopa, Jean A van Wyk, Lewis McCurdy, Gregory P Fusco, Michael B Wohlfeiler

**Affiliations:** can community health, Miami Beach, FL; AIDS Healthcare Foundation\ NYU School of Medicine, New York, New York; Epividian, Inc., Durham, North Carolina; Epividian, Inc., Durham, North Carolina; Aids Healthcare Foundation, Miami Beach, Florida; AIDS Healthcare Foundation, Los Angeles, California; ViiV Healthcare, Fairfax, Virginia; ViiV Healthcare, Fairfax, Virginia; ViiV Healthcare, Fairfax, Virginia; ViiV Healthcare Limited, Brentford, England, United Kingdom; Atrium Health, Charlotte, North Carolina; Epividian, Inc., Durham, North Carolina; AIDS Healthcare Foundation, Los Angeles, California

## Abstract

**Background:**

The first long-acting (LA) antiretroviral therapy (ART) regimen, cabotegravir+rilpivirine (CAB+RPV) injection, was approved by the FDA in January 2021 for ART-experienced, people with HIV (PWH) with undetectable viral load (VL< 50 copies/mL). We assessed clinical effectiveness of CAB+RPV LA in the first year of use in the United States (US).

**Methods:**

Using electronic health record data from the OPERA® cohort, all ART-experienced adults who received ≥1 CAB+RPV LA prescriptions for the first time between 21Jan2021 and 28Feb2022 were followed until 13Mar2022. Discontinuation was defined as an ART switch or > 2 consecutive missed doses. VL were monitored from first injection until end of follow-up or discontinuation. Confirmed virologic failure was defined as 2 consecutive VLs > 200 copies/mL or 1 VL > 200 copies/mL + discontinuation. Results were stratified by VL at first prescription (i.e., suppressed: < 200 copies/mL; viremic: ≥ 200 copies/mL).

**Results:**

Of 994 PWH prescribed CAB+RPV, all were ART-experienced and 85% had undetectable VL (< 50 copies/mL), 90% were suppressed (< 200 copies/mL), and the remainder had VL ≥200/mL (6%) or missing baseline VL (4%). Of those prescribed, 344 (38%) received CAB+RPV LA injections over a median 53 (IQR: 35, 79) days; 14% were women, 36% were Black, 29% were Hispanic, 25% had a BMI of ≥30, and the median age was 40 (IQR: 32, 53) years (Table 1). At the end of observation, 62% had not yet received CAB+RPV injections as they were in the process of approval, were on oral lead-in, or had been denied. At study end, 310 (90%) of the 344 remained on CAB+RPV LA with median follow-up of 3.4 (2.2, 6.1) months. Among those with VLs after first injection, the last VL was < 200 copies/mL in 99% and < 50 copies/mL in 94% (Table 2); all follow-up VLs were < 200 copies/mL in 97%, and < 50 copies/mL in 88%. Thirty viremic PWH received CAB+RPV LA injections (Table) with a median VL at first prescription of 4.2 (IQR: 3.2, 4.9) log copies/mL. Five or fewer PWH experienced confirmed virologic failure in each of the suppressed and viremic groups.

**Conclusion:**

In this real-world cohort of PWH who received CAB+RPV LA injections in the US, observations from the first year suggest that this regimen is effective among virologically suppressed individuals.

**Disclosures:**

**Michael G. Sension, MD**, Gilead: Advisor/Consultant|Gilead: Honoraria|Viiv: Advisor/Consultant|Viiv: Grant/Research Support|Viiv: Honoraria **Ricky K. Hsu, MD**, Gilead: Honoraria|Merck: Honoraria|ViiV: Advisor/Consultant|ViiV: Grant/Research Support|ViiV: Honoraria **Jennifer S. Fusco, BS**, AIDS Healthcare Foundation: Client of my employer|EMD Serono: Client of my employer|Gilead Sciences: Client of my employer|Janssen: Client of my employer|Merck & Co.: Client of my employer|TheraTechnologies: Client of my employer|ViiV Healthcare: Client of my employer **Laurence Brunet, PhD**, AIDS Healthcare Foundation: Client of my employer|EMD Serono: Client of my employer|Gilead Sciences: Client of my employer|Janssen: Client of my employer|Merck & Co: Client of my employer|TheraTechnologies: Client of my employer|ViiV Healthcare: Client of my employer **Gayathri Sridhar, MBBS, MPH, PhD**, GlaxoSmithKline: Stocks/Bonds|ViiV Healthcare: Employment **Vani Vannappagari, MBBS, MPH, PhD**, ViiV Healthcare: I am full time employee of ViiV Healthcare and receive GlaxoSmithKline stock as part of my compensation package|ViiV Healthcare: Stocks/Bonds **Andrew Zolopa, MD**, ViiV Healthcare: full time employee|ViiV Healthcare: Stocks/Bonds **Jean A. van Wyk, MB,ChB; MFPM**, ViiV Healthcare Limited: I am an employee of ViiV Healthcare|ViiV Healthcare Limited: Stocks/Bonds **Gregory P. Fusco, MD, MPH**, AIDS Healthcare Foundation: Client of employer|EMD: Grant/Research Support|Gilead Sciences: Client of employer|Janssen: Client of employer|Merck & Co.: Client of employer|Theratechnologies: Client of employer|ViiV Healthcare: Client of employer.

